# Systematic Review of the Use of the 6-Minute Walk Test in Measuring and Improving Prognosis in Patients With Ischemic Heart Disease

**DOI:** 10.1016/j.cjco.2023.08.003

**Published:** 2023-08-13

**Authors:** Andrew Coulshed, David Coulshed, Faraz Pathan

**Affiliations:** a Royal Prince Alfred Hospital, Sydney, New South Wales, Australia; b Department of Cardiology, Nepean Hospital, University of Sydney, Penrith, New South Wales, Australia

## Abstract

**Introduction:**

The 6-minute walk test (6MWT) has been used for over 30 years to assess exercise capacity in patients with respiratory disease, and more recently, in those with heart failure. However, despite being a simple and reproducible test of real-world exercise capacity, its use in patients with ischemic heart disease (IHD) is less well accepted. We sought to review systematically the evidence surrounding the 6MWT in IHD.

**Methods:**

We searched the Medline, PubMed, Embase, and Scopus databases for the following key terms: “six minute walk test/6 minute walk test/6MWT” and “angina/coronary artery disease/coronary disease/IHD/ischemic heart disease.” We followed **P**referred **R**eporting **I**tems for **S**ystematic Reviews and **M**eta-**a**nalysis (PRISMA) guidelines to select publications for full-text review and analyzed the collated data.

**Results:**

A total of 1228 unique papers were found, of which 71 were chosen for full-text review and 37 for detailed analysis. Most (23) concerned the effect on 6MWT distance (6MWTd) of cardiac rehabilitation, with measurements commenced after an intervention (acute myocardial infarction, n = 4; open heart surgery (OHS), n = 5; percutaneous coronary intervention (PCI), n = 3; or other, n = 11). The effect on 6MWTd of OHS was investigated in 6 studies and of PCI in one study. The 6MWT is a useful measurement of physical capacity; data are limited on its ability to assess benefit following PCI.

**Conclusions:**

The 6MWT has been studied inconsistently in IHD. The majority of data are on patients before and after CR. Data are limited concerning the effect on 6MWTd of OHS or PCI. The available data support the 6MWT as a measure of change in performance status following coronary intervention. More work is required to confirm this hypothesis.

## Résumé

**Contexte:**

Le test de marche de 6 minutes (TM6) est utilisé depuis plus de 30 ans pour évaluer la tolérance à l’effort chez les patients atteints de maladies respiratoires, et maintenant également utilisé auprès des patients atteints d’insuffisance cardiaque. Toutefois, bien qu’il s’agisse d’un test simple et facile à reproduire de la tolérance à l’effort en contexte réel, son utilisation est moins bien acceptée auprès des patients présentant une cardiopathie ischémique (CI). Notre objectif était de réaliser une revue systématique des données probantes au sujet du TM6 chez les patients atteints de CI.

**Méthodologie:**

Nous avons effectué une recherche dans les bases de données Medline, PubMed, Embase et Scopus en utilisant les mots-clés suivants : « six minute walk test/6 minute walk test/6MWT » [test de marche de six minutes/test de marche de 6 minutes/TM6] et « angina/coronary artery disease/coronary disease/IHD/ischemic heart disease » [angine/coronaropathie/CI/cardiopathie ischémique]. Nous avons suivi les lignes directrices « **P**referred **R**eporting **I**tems for **S**ystematic Reviews and **M**eta-**a**nalysis » pour sélectionner les articles à soumettre à un examen du texte intégral et nous avons analysé les données obtenues.

**Résultats:**

Au total, 1 228 articles différents ont été repérés; 71 d’entre eux ont été sélectionnés pour un examen du texte intégral, puis 37 ont été soumis à une analyse plus détaillée. La plupart de ces articles (23) portaient sur l’effet de la distance au TM6 (dTM6) sur la réadaptation cardiaque, et les mesures débutaient après une intervention (infarctus aigu du myocarde, n = 4; chirurgie à cœur ouvert [CCO], n = 5; intervention coronarienne percutanée [ICP], n = 3; autre type d’intervention, n = 11). L’effet sur la dTM6 d’une CCO a été évalué dans six études et l’effet d’une ICP a été évalué dans une étude. La dTM6 est une mesure utile de la tolérance à l’effort, mais les données restent limitées sur sa capacité à évaluer les effets bénéfiques d’une ICP.

**Conclusions:**

L’utilisation du TM6 n’a pas été étudiée de façon systématique en contexte de CI. La majorité des données portent sur les patients avant et après la réadaptation cardiaque, mais il y a peu de données probantes sur l’effet d’une CCO ou d’une ICP sur la dTM6. Les données dont on dispose corroborent l’utilisation du TM6 comme mesure d’un changement des capacités après une intervention coronarienne. D’autres travaux de recherche devront être réalisés pour confirmer cette hypothèse.

Walking tests have a long history of being used to assess exercise capacity. Cooper^1^ introduced a 12-minute walk test to assess oxygen uptake. Although the 12-minute walk test is a useful clinical tool,^2^ many patients with pulmonary disease were unable to complete the test. However, a test of half that length of time was demonstrated to be readily usable to assess patients with pulmonary disease.^3^ The 6-minute walk test (6MWT) has subsequently become well established as a method of estimating prognosis in patients with a wide range of clinical conditions, including pulmonary disease^4^^,^^5^ (especially pulmonary hypertension^6^) and heart failure.^7^ The 6MWT has also been studied as a measure of response to therapy in these conditions—for example, the response to vasodilator therapy for pulmonary hypertension,^8^ or the implantation of biventricular pacemakers in patients with heart failure.^9^ The distance achieved in a 6MWT has been demonstrated to be a good assessment of functional capacity in daily life.^10^ The high reproducibility of the distance walked in a 6MWT (6WMTd) means that even relatively small incremental changes in distance have been demonstrated to show a significant change in functional capacity.^11^

Equally, demand remains for a well-validated tool to assess prognosis and response to therapy in ischemic heart disease. In the era of the **C**linical **O**utcomes **U**sing **R**evascularization and **A**ggressive Dru**g E**valuation (COURAGE),^12^ **I**nternational **S**tudy of **C**omparative **H**ealth **E**ffectiveness With **M**edical and **I**nvasive **A**pproaches (ISCHEMIA),^12^ and, **O**bjective **R**andomised **B**linded **I**nvestigation With Optimal Medical **T**herapy of **A**ngioplasty in Stable Angina (ORBITA)^13^ trials, we believe that a considerable need exists for a test that reflects the real-world patient response to coronary interventions. The 6MWT is able to do this in other cardiac-related situations, and we believe that it could be applied to coronary artery interventions if it can be shown to be both reproducible and responsive to reductions in cardiac ischemia. Broadly, although symptom-limited treadmill-based exercise testing has been demonstrated to reflect prognosis,^14^, ^15^, ^16^ once a treadmill-based exercise test progresses beyond 7 metabolic equivalents (METS), at the second stage of the Bruce protocol, it imposes physiological demands that are in excess of usual day-to-day requirements, and beyond this stage, many patients simply cannot keep up with the speed and gradient of the treadmill. In the ORBITA study,^13^ Bruce protocol treadmill testing was unable to demonstrate any response to percutaneous coronary intervention (PCI), using detailed analysis of electrocardiogram changes and oxygen consumption at supra-normal exercise levels. Quantification of symptoms using more typical activity–related symptoms was not given.

In comparison, the 6MWT reflects an exertion level similar to that required for a patient’s usual activities, and it has been demonstrated to correlate strongly to outdoor walking capacity.^10^ Additionally, in patients with heart failure, the 6MWT has been demonstrated to have a high degree of reproducibility^17^^,^^18^—the test requires little more than personnel familiar with the technique and an unobstructed 25-metre length of flat ground. The physiological demands of the 6MWT remain clinically significant and comparable to those of exercise testing. Patients reach comparable exertion levels in the 2 tests, as measured through heart rate, maximal ventilation, and blood pressure.^19^ More broadly, cardiorespiratory fitness has been well demonstrated to reflect prognosis in patients with cardiorespiratory disease.^20^^,^^21^

Thus, the 6MWT arguably can be used as a safe, noninvasive, and reproducible test to assess response to therapy in patients with ischemic heart disease (IHD), with response measured in terms of both reduced symptoms and improved prognosis. Currently, however, data on the use of the 6MWT in IHD are heterogeneous and varied.

Consequently, we sought to perform a systematic review and narrative analysis of pertinent evidence about the usefulness of the 6MWT in assessing response to any forms of therapy, to improve either symptoms or prognosis in patients with IHD. The key questions, therefore, are as follows: (i) Is the same level of reproducibility found in IHD?; (ii) Does the 6MWTd reflect the response to treatment?; (iii) What is the evidence that the 6MWT result can be used to estimate prognosis, together with its change following intervention?; and (iv) What is the change in 6MWTd following symptomatically successful medical intervention, PCI, or open heart surgery (OHS)?

Evidence is limited that the 6MWT measures prognosis in patients with IHD, such as that in patients after OHS, and in patients who have undergone revascularization following ST-elevation myocardial infarction.^22^ Based on this evidence, we postulated that patients limited in functional capacity by IHD, even in the absence of cardiac failure, will have reduced performance in a 6MWT, and that treatment of cardiac ischemia will improve their 6MWTd.

## Materials and Methods

### Search strategy

This study followed the **P**referred **R**eporting **I**tems for **S**ystematic Reviews and **M**eta-**a**nalysis (PRISMA) guidelines.^23^ We included all studies published concerning patients undergoing treatment for IHD, in which a 6MWT was used to measure either prognosis or changes in symptoms following treatment of IHD. Under the guidance of a librarian trained in conducting systematic reviews, we searched the Medline, PubMed, Embase, and Scopus databases for the following key terms: “six minute walk test/6 minute walk test/6MWT” and “angina/coronary artery disease/coronary disease/IHD/ischemic heart disease.” Further texts were identified through reference list review of selected articles. The search was limited to adult human studies. The search was completed in June 2023.

### Study selection

After duplicates were excluded, a title and abstract review was undertaken, with studies being excluded if the 6MWT was not a key variable measured. Research also was excluded from further consideration if the study population was inappropriate, or if the study was not available in English.

A full-text review was undertaken of the remaining articles. Quality metrics of each included study were assessed according to the Critical Appraisal Skills Programme (CASP) criteria.^24^ Study selection is detailed further in Supplemental Appendix S1.

### Data collection

Two investigators (D.C. and A.C.) collected study characteristics, clinical characteristics, and 6MWT data from individual studies. The quality of studies was assessed using the CASP criteria.^24^ Disagreements were resolved by consensus or in combination with a third investigator (F.P.).

A narrative summary was used to encapsulate the results of the systematic review, as the data were too heterogeneous to allow formal meta-analysis of the 6MWTd data. The review subsequently aimed to summarize the role of 6MWTd as a measure of prognosis and of response to intervention for IHD.

## Results

### Search results

**Figure 1:**
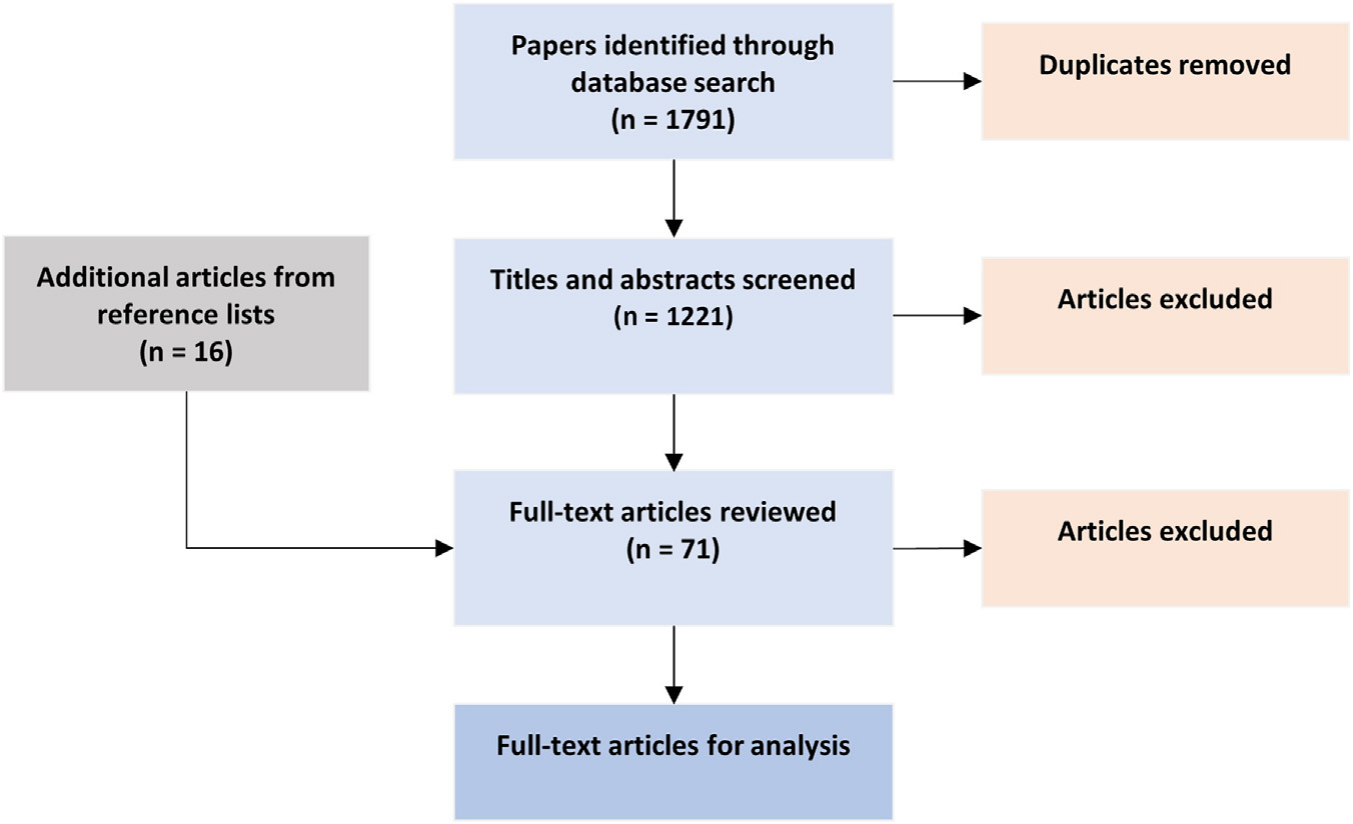
**P**referred **R**eporting **I**tems for **S**ystematic Reviews and **M**eta-**a**nalysis (PRISMA) flowchart of search strategy.

Figure 1 shows the PRISMA flow chart. The initial search strategy revealed 1791 articles. After removing duplicates (570), we scrutinized the titles, removing articles in which the focus clearly either was noncardiac or was on a nonischemic condition, mostly heart failure and valve disease. A total of 55 full-text studies were reviewed, and in reading these and examining their reference lists, 16 additional studies were identified, for a total of 71. However, 34 papers were excluded, as they either failed to address the effect of IHD treatments on 6MWTd and/or prognosis or were of manifestly poor quality. The remaining 37 publications are shown in Table 1.

**Table 1:** Studies included in detailed analysis

Author (year)	Country	Age, y	Gender, M:F	No. of patients undergoing intervention	Study type	Circumstances / population	Initial 6MWTd	6MWTd after intervention	*P*
Araya-Ramirez et al.^46^ (2010)	USA	62 ± 12	70:30	425	Retrospective chart review of responses to CR	Post-OHS (differing types)	399 ± 87	472 ± 97	< 0.001
Babu et al.^47^ (2010)	India	48 ± 11	83:17	15 (and 15 controls)	Retrospective study of response to CR, with matched controls	Post AMI	None	470 ± 152	< 0.01
Baldasseroni et al.^48^ (2016)	Italy	80 ± 4	71:29	160	Prospective cohort study of responses to CR	Older subjects post-AMI or -OHS	398 ± 93	434 ± 92	N/A
Bargehr et al.^49^ (2017)	USA	62 ± 11	74:26	541	Retrospective data review to predict response to CR	Post-OHS (differing types)	52 to 658 (range only)	130 to 743 (range only)	N/A
Beatty et al.^26^ (2012)	USA	67 ± 10	86:14	556	Observational outcome study∗	All types of IHD community patients	87 to 837 (median 480)	None	N/A
Bellet et al.^50^ (2011)	Australia	62 ± 11	74:26	47	Prospective cohort study of responses to CR	All patients entering CR	508 ± 85	532 ± 86	< 0.001
Bellet et al.^51^ (2015)	Australia	63 ± 10	79:21	254 (and 115 fast-track CR)	Nonrandomized, self-selected cohort study of “fast-track” CR	All patients entering CR	500 ± 85 (495 ± 80)	536 ± 91 (528 ± 87)	< 0.001
Bierbauer et al.^52^ (2020)	Switzerland	69 ± 12	64:36	13,612	Randomized prospective cohort study of the predictors of gain in 6MWTd during CR	All patients entering CR	M: 316 ± 141 W: 248 ± 130	435 ± 151 352 ± 134	< 0.001 < 0.001
Busch et al.^53^ (2012)	Germany	79 ± 3	31:69	84 (and 89 controls)	Randomized prospective cohort study of the effect of CR	4 wk post-OHS	296 ± 84	363 ± 86	0.003
Cacciatore et al.^25^ (2012)	Italy	63 ± 9	85:15	883	Observational prognostic cohort study∗	CR post-OHS	302 ± 102	394 ± 96	< 0.001
Compostella et al.^54^ (2016)	Italy	61 ± 13	82:18	154 (30 with MACE)	Retrospective outcome study	Post-AMI	449 ± 113 (380 ± 115)	513 ± 119 (421 ± 125)	N/A
Dasari et al.^55^ (2020)	USA	65 ± 9	98:2	172	Prospective observational study∗	2 wk post-PCI	None	319	N/A
Ferratini et al.^33^ (2012)	Italy	67 ± 11	70:30	223	Prospective observational cohort	9 d post-OHS	279 ± 95	386 ± 91	< 0.0001
Fiorina et al.^56^ (2007)	Italy	65 ± 11	71:29	348	Prospective observational cohort	15 d post-OHS	281 ± 90	411 ± 107	< 0.001
Hassan et al.^36^ (2014)	Egypt	61 ± 11	72:28	100	Prospective observational cohort	Post-AMI	Median 370 range 162–462	None	N/A
Hayta & Korkmaz^57^ (2017)	Turkey	57 ± 5	52:48	56	Prospective observational cohort	6 wk post-OHS	449 ± 171	554 ± 149	< 0.05
Karaszewski^58^ (2014)	Poland	64	Not given	42	Prospective parallel cohorts	2 types of CR post-OHS	Not given	Average 645	N/A
Ko et al.^59^ (2020)	Republic of Korea	60 ± 8	100:0	108	Prospective observational cohort	2 streams of CR post-PCI	546.1 ± 80.3 (short CR) 549.6 ± 67.8 (long CR)	568.7 ± 75.1 (short CR) 580.8 ± 66.6 (long CR)	< 0.05 < 0.05
La Rovere et al.^39^ (2015)	Italy	66 ± 11	77:23	284	Retrospective Data review∗	15 d post-OHS	248 ± 98	374 ± 107	N/A
Listerman et al.^60^ (2011)	USA	52 ± 11	71:29	794	Prospective observational cohort	Diverse presentations of IHD	No absolute data	No absolute data	N/A
Mandic et al.^61^ (2013)	New Zealand	72 ± 6	66:34	58	Prospective observational cohort†	“Self-reported” IHD	No absolute data	No absolute data	N/A
Marazia et al.^62^ (2015)	Italy	66 ± 11	84:16	81	Randomized cohorts of different drug regimens	10 d post-OHS	199 ± 73	313 ± 46	N/A
Matos-Garcia et al.^63^ (2017)	Brazil	56 ± 10	71:29	23 usual care 31 additional CR	Randomized trials of different CR regimens	Selected patients entering CR	461 ± 74 (469 ± 62)	503 ± 63 (533 ± 41)	< 0.05
De Miranda Silva Nogueira et al.^64^ (2006)	Brazil	54 ± 8	92:8	25	Observational cohort study	1 wk post-AMI	521 ± 64	None	N/A
Oerkild et al.^65^ (2011)‡	Denmark	75 ± 6	60:40	75	Prospective parallel cohorts	Patients entering 2 types of CR	335 ± 120	3-mo increment 17–36 m	N/A
Oerkild et al.^38^ (2012)‡	Denmark	77 ± 7	83:17	40	Prospective parallel cohorts	Patients entering 2 types of CR	308 ± 102	3-mo increment 10–36 m	N/A
Oliveira et al.^66^ (2014)	Brazil	52 ± 13	44:56	60	Observational study of the determinants of 6MWTd	11 d post-OHS	No data	260 ± 89	N/A
Peixoto et al.^67^ (2015)	Brazil	56 ± 4	70:30	43 usual care 45 additional CR	Prospective parallel CR cohorts	Selected patients entering CR post-AMI	439 ± 78 (434 ± 86)	452 ± 111 (520 ± 79)	< 0.001
Rossello et al.^28^ (2016)	Spain	62 ± 10	89:11	47	Prospective cohort study	Pre- and post-PCI for CTOs	417 ± 126	463 ± 103	0.002
Salzwedel et al.^68^ (2016)	Germany	52 ± 7	87:13	397	Retrospective chart review	Selected patients entering CR after PCI or OHS	397 ± 74	497 ± 78	N/A
Savci et al.^69^ (2011)	Turkey	60 ± 9	88:12	50	Subgroup analysis of randomized trial	Pre- and post-OHS for coronary disease	337 ± 65	373 ± 56	< 0.001
Sawatzky et al.^70^ (2014)	Canada	64 ± 8	80:20	15	Prospective parallel cohorts	Pre- and post-OHS for coronary disease	340 ± 60	357 ± 27	N/A
Schofield et al.^35^ (1999)	United Kingdom	60 ± 8	90:10	188	Prospective parallel cohorts	Patients having TMLR	12MWT	Distances not given	N/A
Verrill et al.^71^ (2003)	USA	61 ± 10	67:33	630	Retrospective cohort study	Patients entering various CR programs	379 ± 92	437 ± 91	< 0.001
Wright^72^ (2001)	United Kingdom	62 ± 9	66:34	159	Cohort with later nonmatched controls	Patients entering CR program	317 ± 79	369 ± 81	< 0.001
Yazdanyar et al.^27^ (2014)	USA	77	35:65	1665	Prospective cohort study∗	“Community-dwelling adults” with heart disease	Data in quintiles	Data in quintiles	N/A
Zhang et al.^73^ (2018)	China	70	87:13	65 CR 65 controls	Prospective parallel study of community-based CR	Post-AMI	239 ± 75 (230 ± 77)	413 ± 74 (302 ± 102)	< 0.05

6MWTd, 6-minute walk test distance; 12MWT, 12-minute walk test; AMI acute myocardial infarction; CR, cardiac rehabilitation; CTO, chronic total occlusion; IHD, ischemic heart disease; M:F, male:female ration; MACE, major adverse cardiovascular event; N/A, not applicable; OHS, open heart surgery; PCI, percutaneous coronary intervention; TMLR, transmyocardial laser revascularization.

∗ Study demonstrates that 6MWTd is a predictor of long-term prognosis.

† Study demonstrates a link between 6MWTd and maximal oxygen consumption.

‡ Longer-term studies of 6MWTd after CR is completed. In Oerkild et al.^65^, a 3-month increment of 17–36 metres becomes a 12-month loss of 27–45 metres. In Oerkild et al.^38^, a 3- month increment of 10–36 metres becomes a 12-month loss of 51–55 metres.

Of the papers remaining, most concerned the beneficial effect on 6MWT performance of cardiac rehabilitation (CR) following a variety of interventions or events. Of these, only 6 measured the effect of OHS on 6MWTd, and only a single study investigated the effect of PCI on 6MWTd (in patients undergoing PCI for chronic total occlusions). No studies measured the effect on 6MWTd of medical therapy. In 6 studies, an effort was made to correlate 6MWT results with prognosis.

### Study characteristics

Of the 37 studies chosen for detailed analysis, the size of the study varies from 23 to 1665 subjects (excluding one meta-analysis about risk factors), but more than half have less than 100 patients. Male sex predominates, as reflects the prevalence of IHD. Most are prospective cohort studies (10 are retrospective data reviews), without any form of control group. A total of 23 of the papers measure response to CR in differing settings or with different regimens of exercise. Few examine the effect on 6MWTd of cardiac events, and very few attempt to determine the long-term benefits of CR. Several initially measure 6MWTd in the immediate period of recovery from OHS.

Some longer-term studies of prognosis were included (for example, Cacciatore et al.^25^, Beatty et al.^26^, and Yazdanyar et al.^27^), but these papers do not measure the effects of treatment methods, or correlate changes in 6MWTd to changes in prognosis.

### Quality of studies

The overall quality of the 36 selected studies was variable (an average score of less than 4 using the CASP criteria; Supplemental Table S1). Most commonly, points were lost on account of no long-term follow-up being conducted beyond the intervention (n = 25), failure to identify confounders (n = 19), and inadequately described or biased selection protocols (n = 18). More than half (21) measured the response to CR, but little or no evidence indicates that this measure is a satisfactory surrogate for prognosis.

### 6MWT results in differing clinical scenarios

Studies have investigated the use of the 6MWT for patients with IHD in the context of the recovery after acute myocardial infarction (AMI; n = 6) or OHS (n = 9), the response to PCI (n = 2), or the response to CR, after a variety of interventions (n = 13).

Studies concerning the benefits of a CR program are shown in Table 2. Five of these are post-AMI studies, shown last in Table 2. The other papers concern patients with a variety of cardiac conditions, some following OHS (for a variety of indications), and some following PCI. For the 5 studies of post-AMI patients in which data are available prior to and after CR, the weighted mean 6MWTd improves from 379 ± 105 to 443 ± 97 metres.

**Table 2:** Publications comparing 6-minute walk test results before and after CR

Study author (year)	n	6MWTd before CR, m	SD	6MWTd after CR, m	SD	*P*
Wright et al.^72^ (2001)	159	315	76	377	79	< 0.001
Verrill et al.^71^ (2003)	630	379	92	437	91	< 0.001
Fiorina et al.^56^ (2007)	348	281	90	411	107	< 0.001
Araya-Ramirez et al.^46^ (2010)	425	399	87	472	97	< 0.001
Bellet et al.^50^ (2011)	44	507	85	532	86	< 0.001
Busch et al.^53^ (2012)	141	304	62	357	64	0.003
Cacciatore et al.^25^ (2012)	883	302	102	393	96	< 0.001
Ferratini et al.^33^ (2012)	223	279	95	386	91	< 0.0001
Sandercock et al.^74^ (2013)	54	279	144	329	148	U/A
Bellet et al.^51^ (2015)	369	498	83	534	90	0.001
La Rovere et al.^39^ (2015)	284	248	98	374	107	U/A
Marazia et al.^62^ (2015)	81	199	73	313	46	U/A
Baldasseroni et al.^48^ (2016)	160	398	93	434	92	U/A
Salzwedel et al.^68^ (2016)	489	396	73	495	77	U/A
Hayta & Korkmaz^57^ (2017)	52	449	171	554	149	< 0.05
Babu et al.^47^ (2010)∗	30	379	171	470	152	< 0.01
Compostella et al.^37^ (2017)∗	186	441	120	513	113	U/A
Matos-Garcia et al.^63^ (2017)∗	54	421	108	477	50	< 0.05
Peixoto et al.^67^ (2015)∗	43	253	107	367	64	< 0.001
Zhang et al.^73^ (2018)∗	65	440	154	324	64	< 0.05
Weighted mean	5038	360	95	440	91	—

Distances are presented in metres. Bierbauer et al.^52^ and Ko et al.^59^ are not presented, given that no overall data were available when comparing different streams of CR in one study.

6MWTd, 6-minute walk test distance; CR, cardiac rehabilitation; SD, standard deviation; U/A, unavailable.

∗ Study performed after acute myocardial infarction.

Few data concern the effect of coronary artery bypass grafting (CABG)on the exercise capacity of patients without heart failure (Table 3). Six studies aim to investigate the benefit of CABG in terms of improved exercise capacity as measured by the 6MWT, but only 2 of these have assessments before and after surgery, as shown in Table 3. The other 4 studies form a subset of those shown in Table 3.

**Table 3:** Publications comparing 6-minute walk test results before and after cardiac surgery

Study author (year)	n	6MWTd before CABG	SD	6MWTd post-CABG	SD	6MWTd post–CABG and CR	SD	*P*
Busch et al.^53^ (2012)	141	—	—	304	62	357	64	0.003
Cacciatore et al.^25^ (2012)	883	—	—	302	102	393	96	< 0.001
Fiorina et al.^56^ (2007)	348	—	—	281	90	411	107	< 0.001
Ferratini et al.^33^ (2012)	223	—	—	279	95	386	91	< 0.0001
Weighted mean	1595	—	—	294	96	393	95	
Savci et al.^69^ (2011)	43	337	65	373	56	—	—	< 0.001
Sawatzky et al.^70^ (2014)	15	340	60	357	27	—	—	U/A

Distances are presented in metres. Studies are grouped as follows: the first 4 present solely postsurgical data; the last 2 present the effect of surgery on 6MWTd.

6MWTd, 6-minute walk test distance; CABG, coronary artery bypass grafting; CR, cardiac rehabilitation; SD, standard deviation; U/A, unavailable.

As with the studies concerning cardiac rehabilitation in general, this group has considerable heterogeneity; of most importance is the fact that the first post-surgery 6MWTs were done at differing intervals after the surgery (from 2 to 20 days). In the initial recovery period after the surgery, exercise capacity is going to be limited, predominantly by postoperative pain and other debilitating effects of the operation itself.

Data concerning the improvement in exercise capacity to be expected after PCI is almost completely absent. Rossello et al.^28^ studied 47 patients undergoing coronary intervention for chronic totally occluded coronary arteries, and they found an improvement in 6MWT performance, from 417 ± 126 to 463 ± 103 metres (*P* = 0.002). No data show a link between an improvement in 6MWTd and an improvement in prognosis.

## Discussion

Broadly, the presented data demonstrate the heterogeneous nature of current research into the 6MWT. Within these studies, the 6MWT has been correlated, successfully but variably, with improvements in both exercise capacity and prognosis following interventions for IHD.

6MWTd correlates well with self-reported exercise tolerance.^29^ In this study, good correlation was seen between 6MWTd and both the Duke Activity Status Index and the Short-Form, 36-item Health Survey. Some studies have also shown that 6MWTd correlates with maximum oxygen consumption^30^ and is a good measure of functional capacity in a number of different conditions.

### Prognosis as estimated by the 6MWT

Previous studies have shown that exercise capacity predicts prognosis. Myers et al. demonstrated a 12% reduction in mortality per 1-MET increase during exercise testing.^31^^,^^32^ However, no study has directly demonstrated a reduction in mortality with increased 6MWTd in patients with cardiac ischemia.

### 6MWT after coronary interventions

In contrast to the population suffering from heart failure, few studies have been conducted on patients with cardiac ischemia in which prognosis has been compared to 6MWTd. Data are lacking on the effect of PCI on 6MWT performance. Rossello et al.^28^ performed the 6MWT before and after PCI for chronically occluded coronary arteries. They demonstrated a statistically significant increase in 6MWTd, but information about possible long-term benefit was not presented.

### 6MWT following CABG

Although CABG surgery improves prognosis in certain subjects, little information is available on the effect of such surgery on 6MWTd. General cardiopulmonary recovery from OHS can have an effect on exercise tolerance, and improvement in 6MWTd after early commencement of CR postoperatively is to be expected.^33^ In the study by Ferratini et al.,^33^ subsequent major adverse cardiac events (MACEs) were followed, but data with respect to 6MWT performance are lacking.

Data on the effect of OHS on 6MWTd are very limited. Cacciatore et al.^25^ followed 823 patients after cardiac surgery, but these data are exclusively postoperative and are inconclusive about the benefits of CR. Bargehr et al.^34^ presented a retrospective series in which they state that patients improved more with CR after OHS than after PCI, but no data are presented to show this. Other studies are too small or retest too soon after OHS for the data to be meaningful.

### 6MWT to assess the benefit of PCI

Data are lacking on the effect of PCI on 6MWT performance. Rossello et al.^28^ performed the 6MWT before and after PCI for chronically occluded coronary arteries. They demonstrated a statistically significant increase in 6MWTd, but possible long-term benefit information was not presented.

The recent ORBITA study^13^ did not present collected 6MWTd data, and a study of transmyocardial laser revascularization did not provide numerical data.^35^

### 6MWT after AMI

A number of studies have assessed exercise capacity after AMI using the 6MWT. Hassan et al.^36^ assessed prognosis using the 6MWT in a group of 100 patients after AMI treated with thrombolysis and demonstrated an inverse relationship between 6MWTd and the likelihood of a MACE. Compostella et al.^37^ followed 184 patients following AMI treated with PCI and demonstrated a higher rate of MACEs in those with a shorter 6MWTd.

### Use of 6MWT to assess CR results

Some studies have looked at the effect of CR after particular interventions, but most investigate the effect of CR in a general population with IHD. The assessment of benefit has no uniformity. Several studies demonstrated ongoing improvement in 6MWTd following CR, but these results are inconsistent, as Oerkild et al.^38^ reported a reduction of 6MWTd following cessation of the program, thereby suggesting that performance reflects level of fitness.

Larger studies, not necessarily about particular interventions, appear to show a better long-term outlook in those patients who have the greatest 6MWTd after CR.^26^, ^27^, ^39^

### Summary

6MWTd reflects exercise tolerance well, and it arguably correlates well with both maximal oxygen consumption and functional capacity. However, in the field of symptomatic cardiac ischemia, as can be seen, few studies clearly link 6MWT performance, response to therapy, and prognosis. The majority of the data are from studies in which 6MWT results are compared before and after CR. The 2 main issues with this approach are that the fact that physical ability improves with structured rehabilitation is hardly surprising, and a correlation of these improvements in walking distance with improvements in prognosis has not been directly demonstrated.

Although relief of exertion-related symptoms is a clear aim of interventional practitioners and cardiac surgeons, and the 6MWT appears to be a good measure of “real-life” exercise tolerance, available data are very limited about response to anti-anginal therapy using the 6MWT.

### Future study

The 6MWT is a reliable, reproducible, and easily performed test to assess response to interventions in patients with cardiac disease. The test has been used as a surrogate for measurement of prognosis after a variety of interventions for cardiac ischemia, although no direct evidence supports the use of this approach. Evidence does indicate that 6MWTd can be translated into a measurement of maximal oxygen consumption (VO2 max),^40^^,^^41^ which is itself a proven predictor of prognosis. Given that the 6MWT can be so readily performed, the need is clear for studies aiming to demonstrate directly both that interventions for cardiac ischemia improve the functional status shown by the 6MWT and that these improvements correlate with improvements in longer-term prognosis. These studies then could guide exercise prescription and monitoring of patient progress in real-world practice.

### Limitations

Although we applied the published CASP criteria, we acknowledge that some of these are subjective. Good-quality studies investigating the use of the 6MWT to assess the response to therapies in patients with IHD are lacking. Some research projects were randomized controlled studies, but the comparisons concerned aspects of assessment that had no bearing on treatment response or prognosis, so for the purposes of this review, they are treated as cohort studies.

Additionally, although the above data demonstrate many positives of the 6MWT, its use has certain limitations. Most notably, the test does not involve routine electrocardiographic monitoring, thus limiting its value for diagnosing ischemia or arrhythmia, compared to alternatives. Additionally, as with any test that relies on patient participation, intensity of effort from patients is variable, and so some studies have found that 6MWT results correlate only modestly with VO2 max.^42^ A point worth noting, however, is that although stress testing with peak VO2 measurements may provide the best evidence for predicting prognosis in CR,^43^ recent research has demonstrated the prognostic value of estimated METS alone.^44^ Finally, the 6MWT requires both trained professionals to conduct the test in a standardized manner and the availability of suitable 25-metre spaces. Lower-demand treadmill tests such as the Naughton protocol^45^ also offer less-strenuous alternatives to standard Bruce-protocol stress testing, although the 6MWT retains the aforementioned advantages of a low equipment requirement and its basis in everyday activity.

Finally, this study is limited by the small amount of data concerning the response of 6MWTd to coronary interventions, which makes formal meta-analysis inappropriate.

## Conclusions

The 6MWT, unlike treadmill-based exercise testing, is not a diagnostic test, but it is a test of real-life exercise capacity validated in a scientific manner. Additionally, the 6MWT is simple, reproducible, and acceptable to patients. Consequently, the 6MWT could be used to determine clinical response to cardiac interventions and is likely a good a marker of prognosis. Currently, however, data in this area remain limited and heterogeneous, and thus, large prospective controlled trials are necessary to confirm the utility of the 6MWT.
