# Supplementary material for: Systematic Review of the Use of the 6-Minute Walk Test in Measuring and Improving Prognosis in Patients With Ischemic Heart Disease
Source: CJC Open. 2023 Aug 13;5(11):816–25. doi: 10.1016/j.cjco.2023.08.003 (PMC10679465; doi:10.1016/j.cjco.2023.08.003)
Supplement: Supplementary Material [file mmc1.pdf]

# SUPPLEMENTARY MATERIAL

## Supplemental Appendix S1 – Search Strategy

### Selection of Papers for Review

Initial search strategy produced 1791 results as follows: Medline 168 papers, Pubmed 497 results, Embase 880 results, Scopus 246 results. Of these, simple computerized duplicate searching reduced the number to 1228.

Reading and selection based on titles alone revealed 9 papers where there was strong suspicion that the same data was being presented in a subsequent publication.

307 papers were revealed to have congestive heart failure as the main focus of the publication and these were excluded, together with 51 papers concerning the effects of biventricular pacemakers. 49 papers used the 6MWT to assess lung disease, and 27 pulmonary hypertension. These were not considered further. 52 papers concerned the effects of cardiac valve disease on 6MWT performance. 18 concerned peripheral vascular disease. 20 publications concerned the use of shockwave therapy and counterpulsation, neither of which were considered relevant to this review.

A small number of non-English language articles were found, but simple translations of these sources found them not to be useful. This left 55 publications, which underwent full text review. In reading these publications and scrutinizing their references, a further 16 papers were found justifying full-text review.

## Supplemental Table S1 – CASP Criteria Evaluation for Full-Text Article Reviews

| | Pro- or retro-spective | Use of 6MWT as a quantitative outcome | Number of subjects | CASP criteria score | Comment regarding CASP Assessment |
|---|---|---|---|---|---|
| **Araya-Ramirez**[1] | Retrospective | Yes | 425 | 2/8 | Chart review of selected subjects, incomplete follow-up data. |
| **Babu**[2] | Both | Yes | 30 | 1/8 | Retrospective control group with incomplete matching. |
| **Baldasseroni**[3] | Prospective | Yes | 160 | 6/8 | Many exclusion criteria |
| **Bargehr**[4] | Retrospective | Yes | 541 | 7/8 | Retrospective review of criteria predicting poor exercise capacity. |
| **Beatty**[5] | Prospective | Yes | 556 | 7/8 | A “convenience sample” used. |
| **Bellet 2011**[6] | Prospective | Yes | 47 | 6/8 | Repeatability tested well. |
| **Bellet 2015**[7] | Retrospective | Yes | 620 | 3/8 | Larger cohort. Two types of CR. |
| **Bierbauer**[8] | Retrospective | No | 13,612 | 3/8 | 6MWT used as a discriminator, likely heterogenous cardiac rehab centres |
| **Busch**[9] | Prospective | Yes | 141 | 6/8 | Limited to elderly patients, categorical data only. |
| **Cacciatore**[10] | Prospective | Yes | 883 | 6/8 | Many lost after recruitment. |
| **Compostella 2016**[11] | Retrospective | Yes | 186 | 4/8 | |
| **Compostella 2017**[12] | Retrospective. | Yes | 186 | 7/8 | Patients selected after a “complicated” cardiac event, and follow-up was incomplete. Similar data to prior study |

| | | | | | |
|---|---|---|---|---|---|
| **Dasari**[13] | Prospective | Yes | 176 | 3/8 | 98% male, data not given, 10% dropout rate |
| **Ferratini**[14] | Prospective | Yes | 223 | 5/8 | Correlation of 6MWTd with biomarkers. |
| **Fiorina**[15] | Prospective | Yes | 348 | 7/8 | Excessive losses after recruitment. |
| **Hassan AK**[16] | Prospective | No | 100 | 4/8 | Significant selection bias |
| **Hayta**[17] | Retrospective | Yes | 52 | 2/8 | Selection bias and shortened follow-up |
| **Karaszewski**[18] | Prospective | No | 42 | 2/8 | |
| **Ko**[19] | Prospective | Yes | 193 | 3/8 | Different streams of rehab, unclear what criteria determined which patients were allocated to each stream, all male cohort. |
| **La Rovere**[20] | Retrospective | Yes | 284 | 7/8 | Pre- and post-CR 6MWTs correlated with all-cause mortality |
| **Listerman**[21] | Retrospective | No | 794 | 4/8 | Unclear focus of study |
| **Mandic**[22] | Prospective | No | 58 | 3/8 | Non-standard 6MWT. Recruitment bias. |
| **Marazia**[23] | Prospective | Yes | 81 | 5/8 | Randomised trial of ivrabidine v bisoprolol |
| **Matos-Garcia**[24] | Prospective | Yes | 54 | 3/8 | Some unclearly-justified exclusion criteria |
| **Nogueira**[25] | Prospective | No | 25 | 7/8 | Small group of largely male patients |
| **Oerkild 2011**[26] | Prospective | No | 75 | 6/8 | Home tested against hospital based CR, not randomized. |
| **Oerkild 2012**[27] | Prospective | No | 40 | 3/8 | |
| **Oliveira**[28] | Prospective | No | 60 | 5/8 | Aimed to identify higher-risk patients in a low risk cohort. |

| | | | | | |
|---|---|---|---|---|---|
| **Peixoto**[29] | Prospective | Yes | 88 | 3/8 | Low-risk cohort selected at the start. |
| **Rossello**[30] | Prospective | Yes | 47 | 7/8 | |
| **Salzwedel**[31] | Retrospective | Yes | 489 | 4/8 | 6MWTd as a predictor of Return-to-Work |
| **Savci**[32] | Prospective | Yes | 43 | 5/8 | 6MWTs before and after CAGs |
| **Sawatzky**[33] | Prospective | Yes | 15 | 6/8 | Small study with lots of exclusions |
| **Schofield**[34] | Prospective | Yes | 188 | 7/8 | Not 6MWT but 12MWT. |
| **Verrill**[35] | Retrospective | Yes | 630 | 2/8 | A survey only |
| **Wright**[36] | Prospective | Yes | 159 | 6/8 | |
| **Yazdanyar**[37] | Prospective | Yes | 1665 | 5/8 | Large population study of 6MWTd as a predictor of mortality |
| **Zhang**[38] | Prospective | Yes | 65 | 3/8 | |

**Reference List**


1. Araya-Ramírez F, Briggs KK, Bishop SR, Miller CE, Moncada-Jiménez J, Grandjean PW. Who is likely to benefit from phase II cardiac rehabilitation? Journal of cardiopulmonary rehabilitation and prevention. 2010;30(2):93-100.
2. Babu AS, Noone MS, Haneef M, Naryanan SM. Protocol-Guided Phase-1 Cardiac Rehabilitation in Patients with ST-Elevation Myocardial Infarction in A Rural Hospital. Heart Views: The Official Journal Of The Gulf Heart Association. 2010;11(2):52-6.
3. Baldasseroni S, Pratesi A, Francini S, Pallante R, Barucci R, Orso F, et al. Cardiac Rehabilitation in Very Old Adults: Effect of Baseline Functional Capacity on Treatment Effectiveness. Journal of the American Geriatrics Society. 2016;64(8):1640-5.
4. Bargehr J, Thomas CS, Oken KR, Thomas RJ, Lopez-Jimenez F, Trejo-Gutierrez JF. Predictors of Suboptimal Gain in Exercise Capacity After Cardiac Rehabilitation. The American journal of cardiology. 2017;119(5):687-91.
5. Beatty AL, Schiller NB, Whooley MA. Six-minute walk test as a prognostic tool in stable coronary heart disease: data from the heart and soul study. Archives of internal medicine. 2012;172(14):1096-102.
6. Bellet RN, Francis RL, Jacob JS, Healy KM, Bartlett HJ, Adams L, et al. Repeated six-minute walk tests for outcome measurement and exercise prescription in outpatient cardiac rehabilitation: A longitudinal study. Archives of physical medicine and rehabilitation. 2011;92(9):1388-94.
7. Bellet RN, Francis RL, Adams L, Morris NR. Six-minute walk test distances in fast-track and traditional cardiac rehabilitation: A 3-year database review. Journal of cardiopulmonary rehabilitation and prevention. 2015;35(6):417-22.
8. Bierbauer W, Scholz U, Bermudez T, Debeer D, Coch M, Fleisch-Silvestri R, et al. Improvements in exercise capacity of older adults during cardiac rehabilitation. European journal of preventive cardiology. 2020;27(16):1747-55.
9. Busch JC, Lillou D, Wittig G, Bartsch P, Willemsen D, Oldridge N, et al. Resistance and balance training improves functional capacity in very old participants attending cardiac rehabilitation after coronary bypass surgery. Journal of the American Geriatrics Society. 2012;60(12):2270-6.
10. Cacciatore F, Abete P, Mazzella F, Furgi G, Nicolino A, Longobardi G, et al. Six-minute walking test but not ejection fraction predicts mortality in elderly patients undergoing cardiac rehabilitation following coronary artery bypass grafting. European journal of preventive cardiology. 2012;19(6):1401-9.
11. Compostella L, Lakusic N, Russo N, Setzu T, Compostella C, Vettore E, et al. Functional parameters but not heart rate variability correlate with long-term outcomes in St-elevation myocardial infarction patients treated by primary angioplasty. International journal of cardiology. 2016;224:473-81.
12. Compostella L, Lorenzi S, Russo N, Setzu T, Compostella C, Vettore E, et al. Depressive symptoms, functional measures and long-term outcomes of high-risk ST-elevated myocardial infarction patients treated by primary angioplasty. Internal and emergency medicine. 2017;12(1):31-43.
13. Dasari TW, Patel B, Wayangankar SA, Alexander D, Zhao YD, Schlegel J, et al. Prognostic value of 6-minute walk distance in patients undergoing percutaneous coronary intervention: A veterans affairs prospective study. Texas Heart Institute journal. 2020;47(1):10-4.
14. Ferratini M, Ripamonti V, Masson S, Grati P, Racca V, Cuccovillo I, et al. Pentraxin-3 predicts functional recovery and 1-year major adverse cardiovascular events after rehabilitation of cardiac surgery patients. Journal of cardiopulmonary rehabilitation and prevention. 2012;32(1):17-24.
15. Fiorina C, Vizzardi E, Lorusso R, Maggio M, De Cicco G, Nodari S, et al. The 6-min walking test early after cardiac surgery. Reference values and the effects of rehabilitation programme. European Journal Of Cardio-Thoracic Surgery: Official Journal Of The European Association For Cardio-Thoracic Surgery. 2007;32(5):724-9.

16. Hassan AK, Dimitry SR, Agban GW. Can exercise capacity assessed by the 6 minute walk test predict the development of major adverse cardiac events in patients with STEMI after fibrinolysis? PloS one. 2014;9(6):e99035.
17. Hayta E, Korkmaz Ö. Cardiac rehabilitation increases the reliability of the 6-minute walk test in patients after coronary artery bypass graft surgery. Heart Surgery Forum. 2017;20(6):E247-E51.
18. Karaszewski D. Comparison of two models of hospital rehabilitation in patients after coronary artery bypass grafting. Kardiochirurgia I Torakochirurgia Polska = Polish Journal Of Cardio-Thoracic Surgery. 2014;11(1):86-9.
19. Ko DH, Lee K, Chung J. Effects of long-term and short-term cardiac rehabilitation programs on cardiovascular risk factors and physical fitness after percutaneous coronary intervention. Journal of Men's Health. 2020;16(3):e29-e37.
20. La Rovere MT, Pinna GD, Maestri R, Olmetti F, Paganini V, Riccardi G, et al. The 6-minute walking test and all-cause mortality in patients undergoing a post-cardiac surgery rehabilitation program. European journal of preventive cardiology. 2015;22(1):20-6.
21. Listerman J, Bittner V, Sanderson BK, Brown TM. Cardiac rehabilitation outcomes: impact of comorbidities and age. Journal of cardiopulmonary rehabilitation and prevention. 2011;31(6):342-8.
22. Mandic S, Walker R, Stevens E, Nye ER, Body D, Barclay L, et al. Estimating exercise capacity from walking tests in elderly individuals with stable coronary artery disease. Disability and rehabilitation. 2013;35(22):1853-8.
23. Marazia S, Urso L, Contini M, Pano M, Zaccaria S, Lenti V, et al. The Role of Ivabradine in Cardiac Rehabilitation in Patients With Recent Coronary Artery Bypass Graft. Journal of cardiovascular pharmacology and therapeutics. 2015;20(6):547-53.
24. Matos-Garcia BC, Rocco IS, Maiorano LD, Peixoto TCA, Moreira RSL, Carvalho ACC, et al. A Home-Based Walking Program Improves Respiratory Endurance in Patients With Acute Myocardial Infarction: A Randomized Controlled Trial. The Canadian journal of cardiology. 2017;33(6):785-91.
25. Nogueira PAdMS, Leal ACM, Pulz C, Nogueira IDB, Filho JAO. Clinical reliability of the 6 minute corridor walk test performed within a week of a myocardial infarction. International heart journal. 2006;47(4):533-40.
26. Oerkild B, Frederiksen M, Hansen JF, Simonsen L, Skovgaard LT, Prescott E. Home-based cardiac rehabilitation is as effective as centre-based cardiac rehabilitation among elderly with coronary heart disease: results from a randomised clinical trial. Age and ageing. 2011;40(1):78-85.
27. Oerkild B, Frederiksen M, Hansen JF, Prescott E. Home-based cardiac rehabilitation is an attractive alternative to no cardiac rehabilitation for elderly patients with coronary heart disease: results from a randomised clinical trial. BMJ Open. 2012;2(6).
28. Oliveira GU, Carvalho VO, de Assis Cacau LP, de Araújo Filho AA, de Cerqueira Neto ML, da Silva Junior WM, et al. Determinants of distance walked during the six-minute walk test in patients undergoing cardiac surgery at hospital discharge. Journal of cardiothoracic surgery. 2014;9(1).
29. Peixoto TCA, Begot I, Bolzan DW, Machado L, Reis MS, Papa V, et al. Early Exercise-Based Rehabilitation Improves Health-Related Quality of Life and Functional Capacity After Acute Myocardial Infarction: A Randomized Controlled Trial. Canadian Journal of Cardiology. 2015;31(3):308-13.
30. Rossello X, Pujadas S, Serra A, Bajo E, Carreras F, Barros A, et al. Assessment of Inducible Myocardial Ischemia, Quality of Life, and Functional Status After Successful Percutaneous Revascularization in Patients With Chronic Total Coronary Occlusion. The American journal of cardiology. 2016;117(5):720-6.
31. Salzwedel A, Reibis R, Wegscheider K, Eichler S, Buhlert H, Kaminski S, et al. Cardiopulmonary exercise testing is predictive of return to work in cardiac patients after multicomponent rehabilitation. Clinical Research In Cardiology: Official Journal Of The German Cardiac Society. 2016;105(3):257-67.

32. Savci S, Degirmenci B, Saglam M, Arikan H, Inal-Ince D, Turan HN, et al. Short-term effects of inspiratory muscle training in coronary artery bypass graft surgery: A randomized controlled trial. Scandinavian Cardiovascular Journal. 2011;45(5):286-93.
33. Sawatzky JAV, Kehler DS, Ready AE, Lerner N, Boreskie S, Lamont D, et al. Prehabilitation program for elective coronary artery bypass graft surgery patients: A pilot randomized controlled study. Clinical rehabilitation. 2014;28(7):648-57.
34. Schofield PM, Sharples LD, Caine N, Burns S, Tait S, Wistow T, et al. Transmyocardial laser revascularisation in patients with refractory angina: a randomised controlled trial. Lancet (London, England). 1999;353(9152):519-24.
35. Verrill DE, Barton C, Beasley W, Lippard M, King CN. Six-minute walk performance and quality of life comparisons in North Carolina cardiac rehabilitation programs. Heart & Lung: The Journal Of Critical Care. 2003;32(1):41-51.
36. Wright DJ, Khan KM, Gossege EM, Saltissi S. Assessment of a low-intensity cardiac rehabilitation programme using the six-minute walk test. Clinical rehabilitation. 2001;15(2):119-24.
37. Yazdanyar A, Aziz MM, Enright PL, Edmundowicz D, Boudreau R, Sutton-Tyrell K, et al. Association Between 6-Minute Walk Test and All-Cause Mortality, Coronary Heart Disease-Specific Mortality, and Incident Coronary Heart Disease. Journal Of Aging And Health. 2014;26(4):583-99.
38. Zhang Y, Zhang L, Wang Y, Cao A, Han C, Zhang R. Application of optimized cardiac rehabilitation program in exercise tolerance and quality of life of elderly patients undergoing percutaneous coronary intervention for acute myocardial infarction. International Journal of Clinical and Experimental Medicine. 2018;11(4):4087-93.
